# Facile modification of polycaprolactone nanofibers with egg white protein

**DOI:** 10.1007/s10856-021-06505-x

**Published:** 2021-03-24

**Authors:** Nergis Zeynep Renkler, Emre Ergene, Seyda Gokyer, Merve Tuzlakoglu Ozturk, Pinar Yilgor Huri, Kadriye Tuzlakoglu

**Affiliations:** 1 Department of Polymer Engineering, Yalova University, 77200 Yalova, Turkey; 2 Department of Biomedical Engineering, Ankara University, Ankara, Turkey; 3 Department of Molecular Biology and Genetics, Gebze Technical University, Kocaeli, Turkey

## Abstract

Synthetic polymers remain to be a major choice for scaffold fabrication due to their structural stability and mechanical strength. However, the lack of functional moieties limits their application for cell-based therapies which necessitate modification and functionalization. Blending synthetic polymers with natural components is a simple and effective way to achieve the desired biological properties for a scaffold. Herein, nanofibrous mats made of polycaprolactone (PCL) and egg white protein (EWP) blend were developed and further evaluated for use as a scaffold for tissue engineering applications. Homogeneous distribution of EWP was achieved throughout the nanofibrous mats, as shown by immunohistochemistry. ATR-FTIR analysis and contact angle measurements have further confirmed the presence of EWP on the surface of the samples. The swelling test showed that PCL/EWP nanofibers have higher water uptake than PCL nanofibrous mats. Also, EWP addition on the nanofibrous mats resulted in an increase in the tensile strength and Young’s modulus of the mats, indicating that the presence of protein can greatly enhance the mechanical properties of the mats. A significantly higher, more uniform, and dispersed cell spreading was observed on days 7 and 14 than that on neat PCL mats, demonstrating the importance of providing the required cues for cell homing by the availability of EWP. Hence, EWP is shown to be a simple and low-cost source for the functionalization of PCL nanofibrous mats. EWP is, therefore, a facile candidate to enhance cellular interactions of synthetic polymers for a wide range of tissue engineering applications.

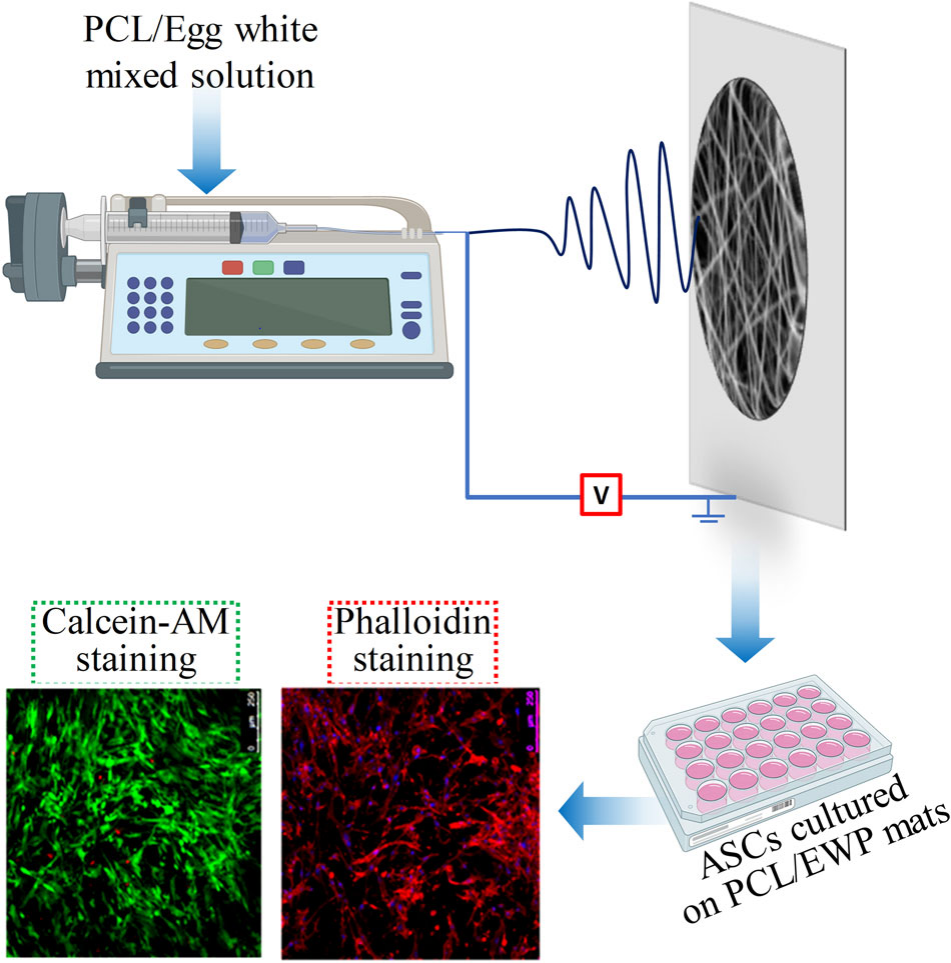

## Introduction

A recognized breakthrough in the field of tissue engineering includes mimicking the natural tissue architecture to achieve the desired cell response for creating 3D tissue equivalents. Among the various processing techniques, electrospinning has received substantial attention in the fabrication of scaffolds for tissue engineering, due to similar structural features of the electrospun nanofibers to the fibrillar component of natural ECM [1, 2]. Nanofibers can be produced at a low cost and simple manner with the electrospinning technique. Furthermore, the ease of incorporation of bioactive compounds into nanofibers is important for producing protein-based biomaterials. In addition, the lack of high temperature during the process is an advantage for the preservation of the activity of sensitive compounds such as proteins [3].

Poly(ɛ-caprolactone) (PCL) is an FDA approved, biodegradable polymer and widely used raw material for scaffold production due to its acceptable biocompatibility, good mechanical properties, and low cost. Its semi-crystalline nature and low melting point allow the fabrication of tissue engineering scaffolds with a variety of structures and forms [4]. However, the hydrophobic nature of PCL has been a major obstacle for initial cell attachment and it even causes a decrease in the proliferation of many cells [5, 6]. To modify this feature of PCL, bulk or surface functionalization strategies can be applied. Several physical and chemical strategies, such as plasma modification, physical adsorption, covalent grafting, chemical etching, have been performed for surface functionalization of PCL nanofibers [7, 8]. Immobilization of bioactive compounds, such as peptides, growth factors, enzymes, polysaccharides, and vitamins, after surface functionalization, promotes the cell/biomaterial interactions [9–13]. Nevertheless, immobilization is usually a multi-step process using different chemicals that may influence the bulk properties of the materials. Also, immobilization efficiency is typically low, therefore a large amount of these delicate and expensive compounds can be wasted. Another drawback of biomolecule immobilization is to control the orientation and concentration of the molecule on the surface, which is a key issue for cell binding [14, 15].

Another way to facilitate interactions between the biomaterial and the cell surface receptors and provide a microenvironment to control cell fate is to fabricate hybrid scaffolds containing a natural polymer/protein. Various studies have been conducted to investigate the potential of nanofibers made of different synthetic–natural polymeric blends [16–19]. This approach is also attempted to use for nanofibrous polycaprolactone substrates to regulate the complex remodeling functions of different cells. Collagen, gelatin, elastin, and silk fibroin are utilized extensively to construct PCL/protein-based matrices for different biomedical applications. For instance, electrospun gelatin/PCL scaffolds are proposed for vascular tissue and guided bone regeneration, among the other applications [20, 21]. Nerve guidance conduits are another application for PCL/protein nanofibers where collagen and elastin have been used to improve the cellular responses [22, 23].

Hen egg white is an inexpensive protein source, containing more than 100 types of soluble proteins with different molecular weights, isoelectric points and concentrations, including ovalbumin (OVA), ovotransferrin, ovomucoid, ovomucin, and lysozyme at high concentrations [24]. Besides being a very good food source, egg white proteins (EWP) provide further benefits for human health, with their antihypertensive, antioxidant, antidiabetic and anticancer features. For instance, the hydrolysate formed by enzymatic hydrolysis of OVA by gastrointestinal protease has been shown to have angiotensin-converting-enzyme (ACE) inhibitor, antihypertensive, antioxidant, and antimicrobial activity [25, 26]. Ovotransferrin is known as, iron-binding protein and can form thermally stable complexes with Fe^3+^, Al^3+^, Cu^2+^, or Zn^2+^ [27]. It also shows antimicrobial and antiviral effects [28]. Ovomucin, which is the main responsible for the white nature of egg white, is a network structure that prevents bacterial movement [24]. It has been reported to inhibit the growth of sarcoma cells and even to cellular damage [29]. Lysozyme, another important egg white protein, has been used extensively as a natural food preservative and has a positive influence on the modulation and stimulation process of the immune system [30]. Moreover, it has been reported that orally administrated lysozyme can inhibit the tumor cells growth, both in vitro and in vivo [31, 32].

This study presents the use of EWP as a simple and accessible source for the modification of synthetic polymers in tissue engineering applications. The electrospinning of PCL polymer with EWP resulted in composite nanofiber mats as a promising matrix for culturing adipose-derived stem cells (ASCs). This proof-of-concept study shows the potential of EWP as a facile source for the modification of polymers to provide cues for cellular attachment and proliferation on synthetic polymers, which can be expanded for various applications in the field of tissue engineering and regenerative medicine.

## Experimental

Hen eggs were obtained from a local supermarket. PCL (Mw = 65000 Da, Sigma Aldrich) was used to produce nanofiber mats. Rabbit anti-ovalbumin antibody was purchased from Merck Millipore (Germany). Alamar Blue cell proliferation assay and Live/Dead kit were acquired from Thermo Fischer (USA). All other chemicals were obtained from Sigma-Aldrich (Germany) and used as received unless otherwise is specified.

### Purification of EWP

EWP was purified from hen eggs according to method described in the literature [33]. Briefly, egg whites were mixed with an equal volume of distilled water and filtered through gauze. The filtrate was stirred magnetically for 1 h in an ice bath and the mixture was then centrifuged at 10,000 × *g* for 20 min at 4 °C. The supernatant was collected and dialyzed against water (MCO = 3500) at 4 °C overnight with four times water change before centrifuging at 15,000 × *g* for 40 min at 4 °C once again. The obtained protein solution was lyophilized for 48 h and stored at 4 °C until use.

SDS-PAGE (12%) was performed using Laemmli method [34]. The gel was stained with Coomassie Brilliant Blue R-250 and destained with 50% ethanol (v/v) and 5% acetic acid. The size of unfolded protein was estimated by comparison to prestained protein molecular weight markers (Thermo Scientific, USA). Bovine serum albumin (66 kDa) was used as a standard protein. Protein concentration was determined by Bradford Assay.

### Production of PCL/EWP nanofibrous mats

For the production of the nanofibrous mats, equal amounts of PCL and EWP were dissolved in 1,1,1,3,3,3-Hexafluoroisopropanol (HFIP) at the final solution concentration of 1 g/ml. The solution was electrospun through a plate collector by applying a high voltage at 19 kV and 20 cm distance between the tip and the collector with a flow rate 1 mL/h.

### Structural and chemical characterization of the PCL/EWP Nanofibrous Mats

The morphology of the nanofibrous mats was determined by a scanning electron microscope (SEM, XL30 ESEM-FEG, FEI-Philips, Netherlands) under 15 kV accelerated electron beam after being vacuum-coated with a thin layer of platinum.

The distribution of EWP on the nanofibrous mats was assessed under a confocal laser scanning microscope (CLSM; Zeiss LSM 880) after immunostaining for ovalbumin. For this, the mats were incubated with the primary rabbit anti-ovalbumin antibody (1:500) for 1 h at RT. The samples were then washed twice with PBS and then incubated for 1 h at RT with the secondary antibody, anti-mouse Alexa Fluor 488 (1:500). Samples were rinsed with PBS before observing with CLSM. To complement the immunostaining results, attenuated total reflection Fourier transform infrared spectroscopy (ATR-FTIR) (FTIR, Perkin Elmer, Spectrum 100, USA) was used to confirm the presence of EWP on the surface of the nanofiber membranes.

Water contact angle (WCA) measurements were performed using distilled water (DI) water at room temperature on the PCL/EWP nanofibrous mats. Samples were placed on a movable stage and leveled horizontally. Approximately 4 μL of DI water was dropped on the surface of the film using a micro-syringe. The contact angles were measured using a Contact Angle Measurement Instrument (CAM 100, KSV) in a conditioned room by recording contact angle values. Measurements were done from 4 different points of each sample.

To determine the final percentage of the components in PCL/EWP nanofibrous mats, thermogravimetric analysis (TGA) was carried out using TA/DTA 6300 Instrument (TA, Instruments, New Castle, DE, USA) under a nitrogen flow, within a temperature range from 25 to 500 °C, and at a scanning rate of 10 °C.min^−1^.

Mechanical properties of PCL/EWP mats and PCL mats were determined using a universal testing machine (Shimadzu AGS-X). Samples (L: 10 mm, H: 40 mm) were elongated under 5 kN load at a speed of 1 mm/min. The applied load and strain of the samples are recorded as a function of time (*n* = 5). Young’s modulus and ultimate tensile strength values of PCL/EWP mats and PCL mats were calculated.

To evaluate water uptake capacity of PCL/EWP nanofibrous mats, 2 × 2 cm pieces of samples were preweighted and immersed in 5 mL of PBS solution (pH = 7.4), then incubated at 37 °C. Samples were taken at certain periods and weighed after wicking the excess fluid with a Kimwipe. The swelling ratio was calculated using the following equation:

$$\left( \% \right) = \frac{{\left( {Ww - Wd} \right)}}{{Wd}} \times 100$$

where W_d_ and W_w_ represents initial dry weight and measured wet weight of samples, respectively. All data are reported as mean ± SD.

In order to investigate in vitro release of EWP from the nanofibrous meshes, samples were cut into 2 × 2 cm pieces, weighed and immersed in 3 mL PBS solution (pH = 7.4) at 37 °C for 30 days. The amount of EWP in the supernatant was assessed by micro-Bradford assay at different time points between days 1–30. At every time point, the collected supernatant was completely replaced with fresh PBS.

### Cell isolation and culture

Adipose-derived stem cells (ASCs) were used as a model cell source to investigate the cellular interactions on PCL/EWP nanofibrous mats. ASCs were selected as they are frequently used in stem cell-based tissue engineering applications, as an abundant, readily available cell source [35–38]. ASCs were isolated from human subcutaneous adipose lipoaspirates under an Institutional Review Board approved (AU Clinical Research Ethical Committee #13-25-15) protocol with informed patient consent. Briefly, the lipoaspirate was washed with equal parts of PBS and digested for 1 h at 37 °C in media containing 1 mg/mL collagenase type I (Worthington Biochemical Corp.), 10 mg/mL bovine serum albumin (Sigma), and 2 mM CaCl_2_ in PBS. After centrifugation and resuspension in a stromal medium composed of DMEM supplemented with 10% FBS, 1% P/S, and 1 ng/mL FGF-2, the stromal vascular pellet was plated. The adherent population (P0) was harvested upon confluence and stored in liquid nitrogen until use [39].

To investigate interactions of ASCs with PCL/EWP nanofibrous mats, samples were sterilized with 70% ethanol for 1 h prior to cell seeding. ASCs at passage 3 were suspended in culture medium (DMEM-high glucose-containing 10% FBS and 1% P/S) and seeded onto scaffolds using a density of 25 × 10^3^ cells/sample and then cultured for 2 weeks at 37 °C in humidified atmosphere containing 5% CO_2_ with medium changes every 2–3 days. Samples were harvested at time points of 2^nd^, 7^th^, and 14^th^ days of culture for cell adhesion and proliferation tests.

### Investigation of cell viability and morphology on the PCL/EWP nanofibrous mats

Cell viability during the 14 days of culture was studied using Live/Dead Kit (Calcein-AM and Ethidium Homodimer-1 (EthD-1) staining) according to the manufacturer’s instructions and were observed with a CLSM (Leica, Germany). Cell viability was further evaluated and quantified using Alamar Blue® (Thermo Fisher Scientific, NY, USA) assay at same time points. The Alamar Blue assay is based on the reduction of blue resazurin dye to red fluorescent resorufin by the mitochondrial enzymes of viable cells. To perform this assay, 1 mL of Alamar Blue dye solution in colorless DMEM (10% v/v) was applied to wells containing samples and allowed to incubate at 37 °C, 5% CO_2_ for 1 h. Following incubation, 200 µL aliquots from the wells were transferred into a 96‐well plate. Absorbance values were measured at 570/590 nm using a microplate reader (Biochrom EZ Read 400, UK). The percentage of reduction was calculated and converted to viable cell numbers using a calibration curve.

In order to investigate cellular organization on the PCL/EWP nanofibrous mats, the actin cytoskeleton of the cells was stained with phalloidin, and nuclei were counterstained with Draq5 on day 14. Briefly, samples were rinsed with PBS and then fixed with 4% paraformaldehyde before incubation with Phalloidin/Draq5 (Phalloidin (1:500, Sigma, USA), Draq5 (1:1000, Thermo Scientific, USA) for 45 min at the dark. Following incubation, samples were visualized by a CLSM (Leica, TCS-SPE). Neat PCL nanofibrous mats were used as a control for all experiments.

Cellular morphology was further examined by SEM. For this, samples were fixed in formaldehyde after washing with cacodylate buffer and dehydrated through graded series of ethanol before drying at room temperature.

### Statistical analysis

All quantitative data from experiments were reported as means±standard deviation for *n* = 6 for each sample. Data were analyzed with statistically significant values defined as *p* < 0.05 based on one-way analysis of variance (ANOVA) followed by Tukey’s test for determination of the significance of the difference between different groups (*p* ≤ 0.05).

## Results and discussion

**Fig. 1:**
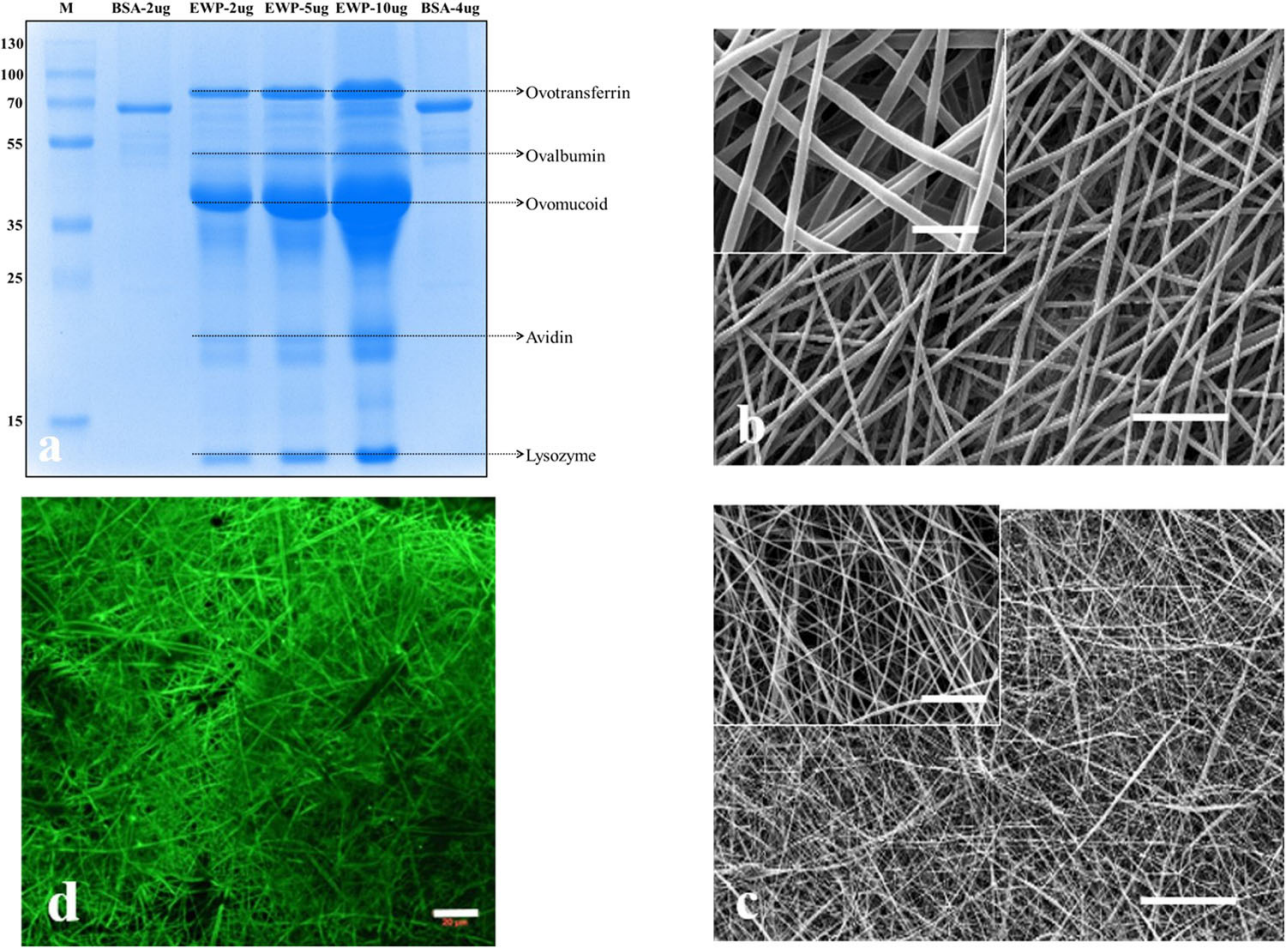
Production of PCL/EWP nanofibrous mats; **a** SDS-PAGE analysis of purified hen egg white; morphology of nanofibrous mats analyzed by SEM, **b** PCL, and **c** PCL/EWP; **d** ovalbumin immunostaining on the surface of PCL/EWP mat

An efficient regeneration in tissue engineering requires a scaffold with proper characteristics in terms of biological and physical performances. Consequently, several processing technologies and engineering strategies have been combined to achieve the desired properties of the scaffolds. Synthetic polymer-based scaffolds can provide favorable structure and mechanical properties but may not be preferable for cellular attachment and/or tissue ingrowth. Unlike synthetic polymers, natural polymers exhibit superior biological properties. However, they lack physical strength and stability in the physiological environment. Thus, the use of composite scaffolds comprised of synthetic and natural components is becoming increasingly common. Herein was proposed the use of EWP for triggering the cell attachment and growth on the nanofibrous scaffolds in which the synthetic component, PCL, meets the physical requirements. The SDS-page analysis given in Fig. 1a exhibited 5 bands corresponding to the main proteins of EWP, which are ovalbumin, ovotransferrin, ovomucoid, avidin, and lysozyme, which are consistent with previous reports [33, 40]. The total protein concentration was calculated to be about 5 mg/ml, which indicates the protein-rich feature of the hen egg white.

Figure 1b and c present the SEM micrographs of nonwoven nanofibrous mat structure produced from equal amounts of PCL/EWP solution via electrospinning process. The collected electrospun PCL/EWP mats were characterized by the absence of beads and exhibited a bimodal fiber distribution which is typical for bicomponent electrospun scaffolds [20, 41]. Mean diameters of neat PCL and PCL/EWP fibers were determined to be 1.182 ± 0.15 μm and 0.167 ± 0.053 μm, respectively. The molecular weight of dissolved material is an important parameter that reflects the solution viscosity. Higher solution viscosity is known to result in more uniform fibers with larger diameters [42, 43]. Thus, blending PCL with EWP, which consists of proteins with a molecular weight lower than PCL, decreased the viscosity of solution though the total concentration was kept the same. Therefore, electrospinning of PCL/EWP solution formed nanofibers with a lower mean diameter than that of neat PCL. It was also observed that the diameter of PCL/EWP nanofibers varied over a wide range, which might be due to the different phase separation behavior of the components and the uncontrollable evaporation of the very volatile solvent, HFIP, during electrospinning [44].

While SEM analysis indicates the formation of smooth fibers for both neat PCL and PCL/EWP mats, perhaps of greater interest is the homogenous distribution and availability of EWP on the fiber surfaces. Therefore, the presence of EWP on the nanofibers was assessed by immunohistochemistry by using antibody raised against ovalbumin. CLSM images, presented in Fig. 1d, confirmed the spatial distribution of ovalbumin through the nanofibers. Blending allows preserving the initial fiber structure characteristic of a scaffold, which is essential to cell proliferation while gathering up the beneficial characteristics of each individual component [45]. Hence, it is superior to other techniques, such as surface modification or coating, which can make destruction or changes of some desired properties of the material through improving another one.

**Fig. 2:**
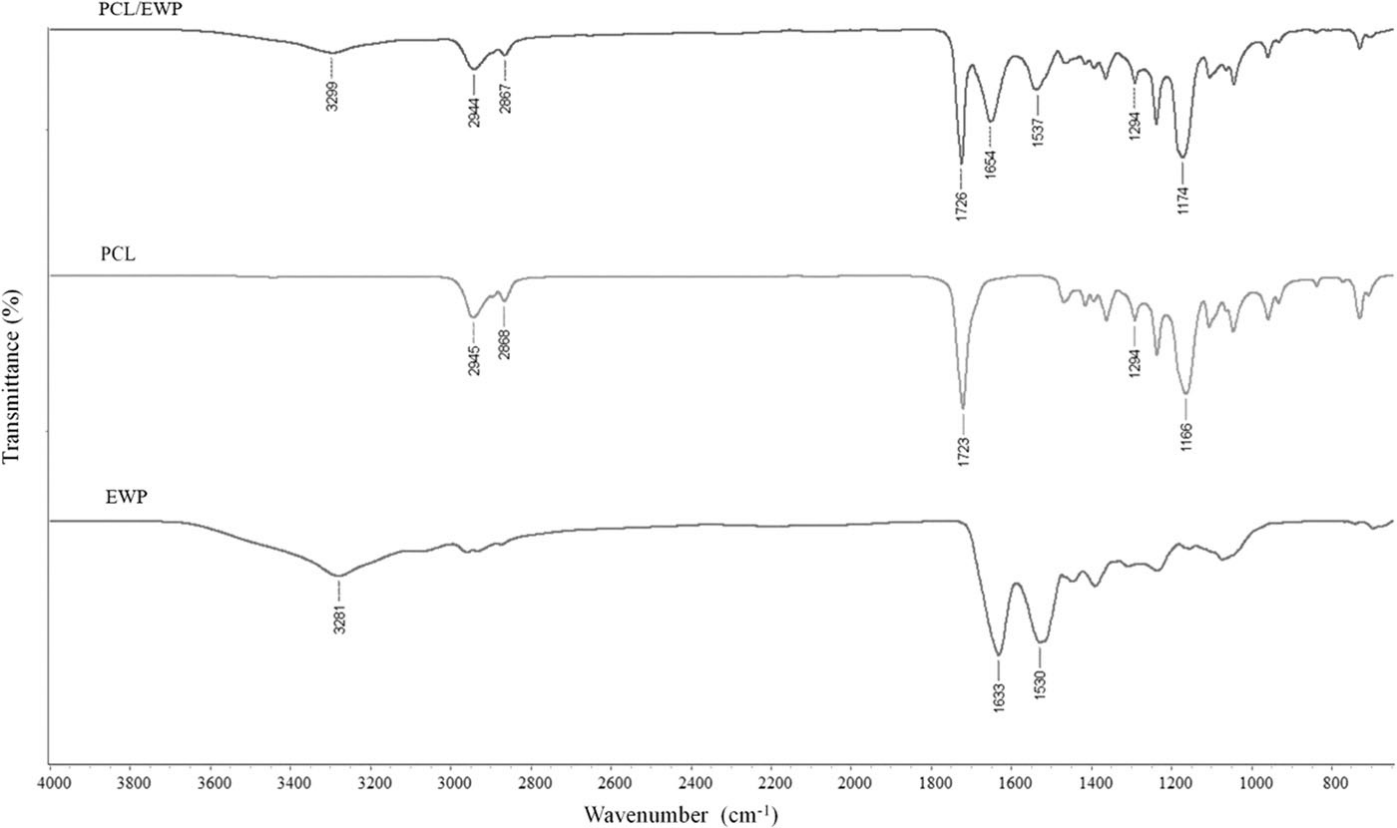
ATR-FTIR spectra of lyophilized EWP, neat PCL, and PCL/EWP mats

Since the surface of the scaffold has primary importance on cell response, it is important to have suitable chemical and topographical cues to bind cell membrane receptors. Thus, ATR-FTIR analysis of the nanofibrous mats was conducted to obtain information regarding the chemical structure of the fiber surfaces. Besides all characteristic peaks of PCL, including carbonyl stretching (1724 cm^−1^), CH_2_ stretching (2867–2944 cm^−1^), C–C, and C–O stretching in the crystalline phase (1293 cm^−1^), and the amorphous band of PCL (1168 cm^−1^), additional bands associated with proteins observed at around 1648 cm^−1^ (amide I) and 1535 cm^−1^ (amide II) in the spectra of PCL/EWP scaffolds (Fig. 2) [46, 47]. -NH_2_ groups are very crucial to bind fibrinogen which plays a vital role for cell adhesion to the biomaterial surfaces [48, 49]. The adsorption of fibrinogen alters integrin binding and subsequent cell adhesive events. Therefore, amine groups on the biomaterial surface can mediate the focal adhesion function and signaling in cell adhesion process [48].

**Fig. 3:**
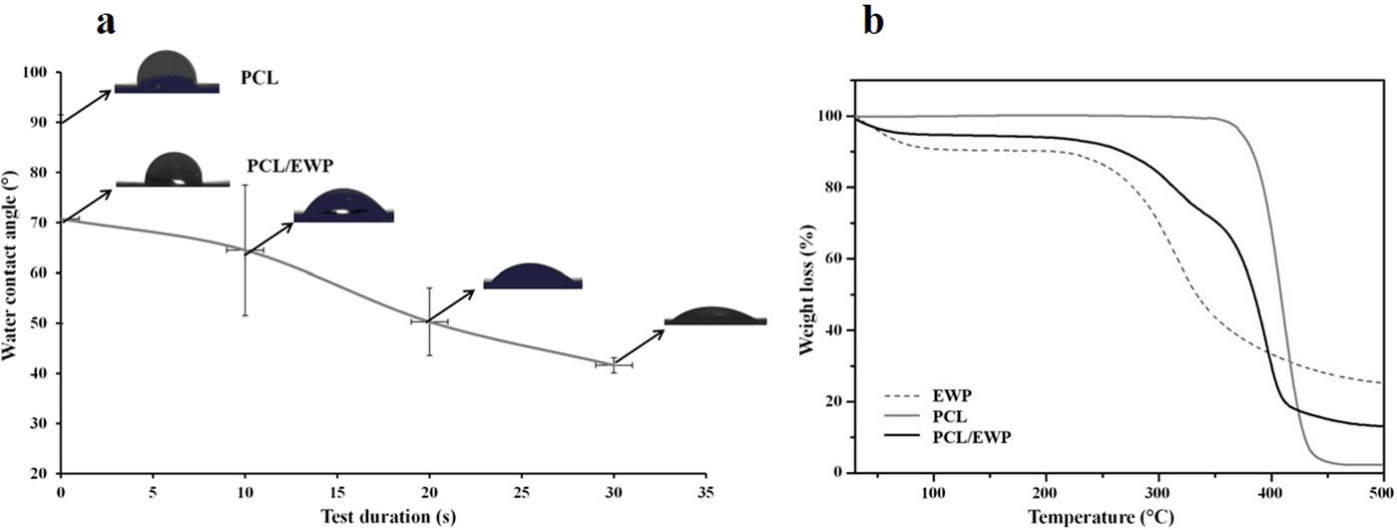
Characterization of PCL and PCL/EWP mats; **a** Water contact angle measurements; **b** TGA thermograms

It has been reported that ASCs preferably adhere to highly hydrophilic and rough surfaces and exhibit broadly stretched morphology compared with that on hydrophobic and smooth surfaces [32]. This might be due to the secondary structure rearrangements of fibronectin, which is more active for cell binding. Considering all these, the WCA measurements were conducted to analyze the wettability of the nanofiber mats. As presented in Fig. 3a, the contact angle on neat PCL mats was found to be 90° which assigned to have a hydrophobic surface and stay constant during the test period. In contrary, PCL/EWP mats showed more hydrophilic character, having a contact angle of 70° at the beginning and decreasing rapidly by absorbance of the water droplet along test time.

The thermal decomposition profiles of PCL, EWP and PCL/EWP analyzed by TGA were presented in Fig. 3b TGA curves of neat PCL showed a one-step thermal decomposition profile at the temperature range 350–439 °C which is consistent with previous reports [50, 51]. Moreover, there was no initial weight loss attributed to water evaporation due to the hydrophobic character of PCL. However, the decomposition of EWP occurred at a much higher temperature range starting from 230 to 358 °C and only 74.5% of the sample was decomposed. There was also an initial weight loss of ca. 9.6% caused by the evaporation of water. In the case of PCL/EWP nanofiber mats, it was possible to observe the decomposition of both components and the calculation of the weight loss of each one. The initial water loss decreased to 5.3% due to the interaction of the components. As the PCL is decomposed around 97.6%, the residual mass can be contributed to EWP. Thus, the final percentage of EWP in the composite nanofibers was found to be around 36%.

Young’s modulus and ultimate tensile strength values of PCL nanofiber and PCL/EWP nanofiber mats are displayed in Table 1. PCL/EWP nanofiber mats reached a maximum tensile strength of 40.92 ± 11.8 MPa, while PCL nanofiber mats 0.97 ± 0.77 MPa. It was also found that Young’s modulus of fiber mats, increasing from 10.75 ± 4.9 for PCL samples to 40.92 ± 11.8 for PCL/EWP samples. Besides the effect of molecular interaction between the protein and the PCL, fiber diameters are also an important factor that might influence the mechanical properties as reported by the others [52, 53]. Consequently, a significant improvement in mechanical properties was achieved by blending PCL with EWP.

**Table 1:** Tensile properties of PCL/EWP mats and PCL mats

Sample code	Young’s modulus	Ultimate tensile strength (MPa)
**PCL/EWP**	40.92 ± 11.80	5.23 ± 1.50
**PCL**	10.75 ± 4.94	0.97 ± 0.77

Since water uptake ability is an important parameter for cell adhesion, swelling tests were performed on the nanofibrous mats. The presence of EWP increases water uptake capacity of the mats from 160% (for PCL mats) to 190% (for PCL/EWP mats) after 1 h soaking in PBS (Fig. S1). The water uptake of nanofiber structures is not only attributed to swelling of nanofibers but also the water entrapped into the pores between fibers. Therefore, it is very difficult to distinguish whether the difference is due to the hydrophilicity of EWP or morphological differences between the two samples. However, the water absorption rate of PCL/EWP mats observed in WCA measurements corroborate the presence of protein might be mainly responsible for the difference between total water uptake values of mats.

The EWP release from the scaffolds in PBS at 37 °C was evaluated by a micro-Bradford assay at specific time points (days 1, 2, 5, 7, 14, 21 and 30). A slight burst release of EWP from PCL/EWP nanofiber mats was observed as approximately 6% of the total EWP during the first 24 h, and no protein was detected in the supernatant in the following time points during 30 days (Fig. S2). Observation of an initial burst release followed by release at lower rate are expected in polymeric nanofibers. Proteins are likely to accumulate on the surface of nanofiber scaffolds composed of protein and polymer blends, and in the early stage, these surface proteins are initially released [54] which was also the case in PCL/EWP mats.

**Fig. 4:**
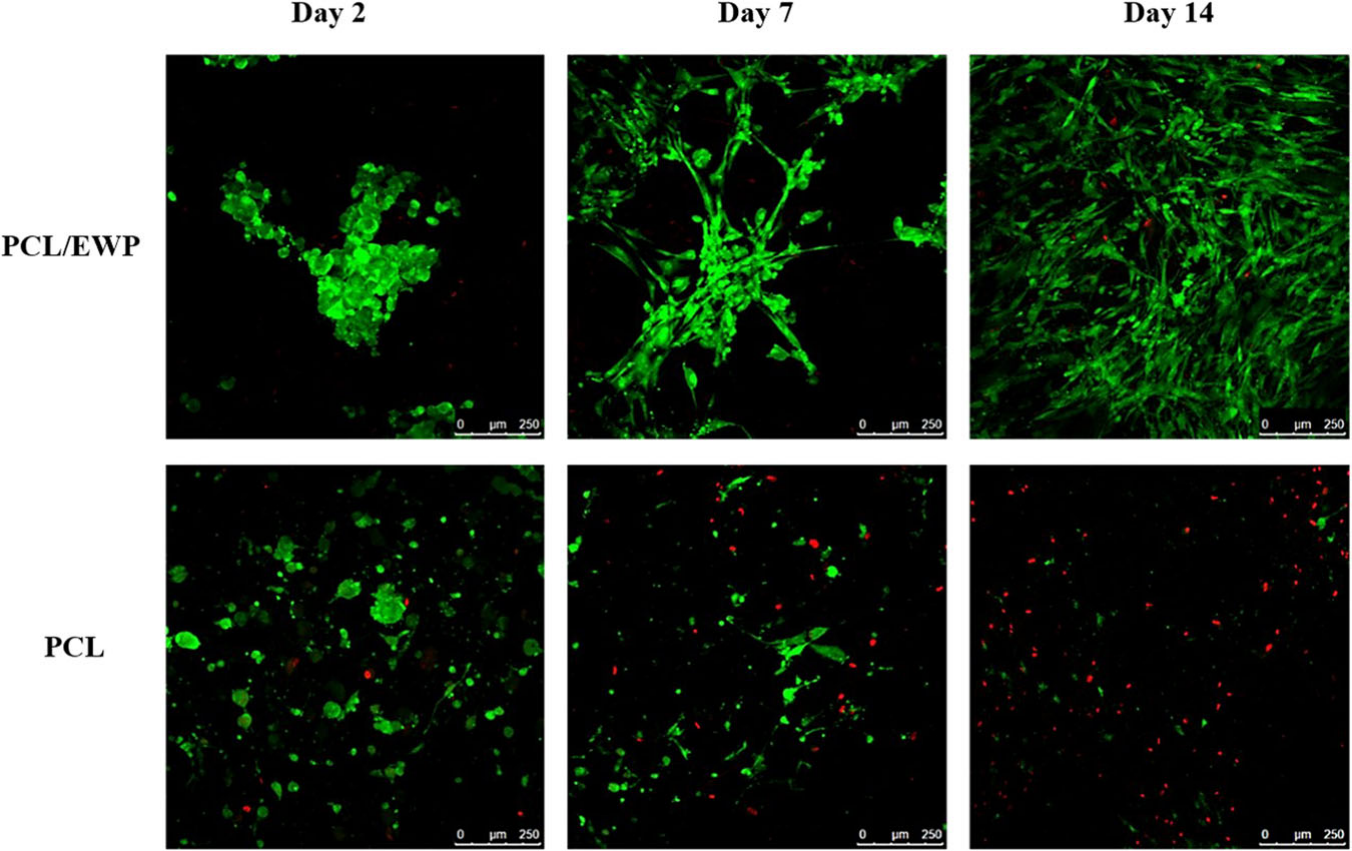
Calcein-AM staining of ASCs seeded on PCL and PCL/EWP nanofibrous mats at 2, 7, and 14 days of culture

The morphology of the ASCs and the extent of cell adhesion and proliferation on the electrospun mats were determined by calcein-AM staining and by its conversion into a green fluorescent and impermeable product by esterases of viable cells, while the EthD-1 stained the dead cells in red. After 2 days, viable cells positively stained with fluorescent dye were widely dispersed all over PCL/EWP mats. Although the morphology of the initial attachment looked similar and even more dispersed on neat PCL mats as compared to PCL/EWP, ASCs were able to spread, elongate and cover the entire surface on PCL/EWP mats for the rest of the culture period with only a few apoptotic cells (Fig. 4). On the contrary, ASCs did not maintain viability and they did not spread on the neat PCL mats as they appeared in red on day 14 (Fig. 4). This shows the importance of adding proper cues on the fiber surfaces using EWP to maintain cell viability and to regulate cellular behavior.

**Fig. 5:**
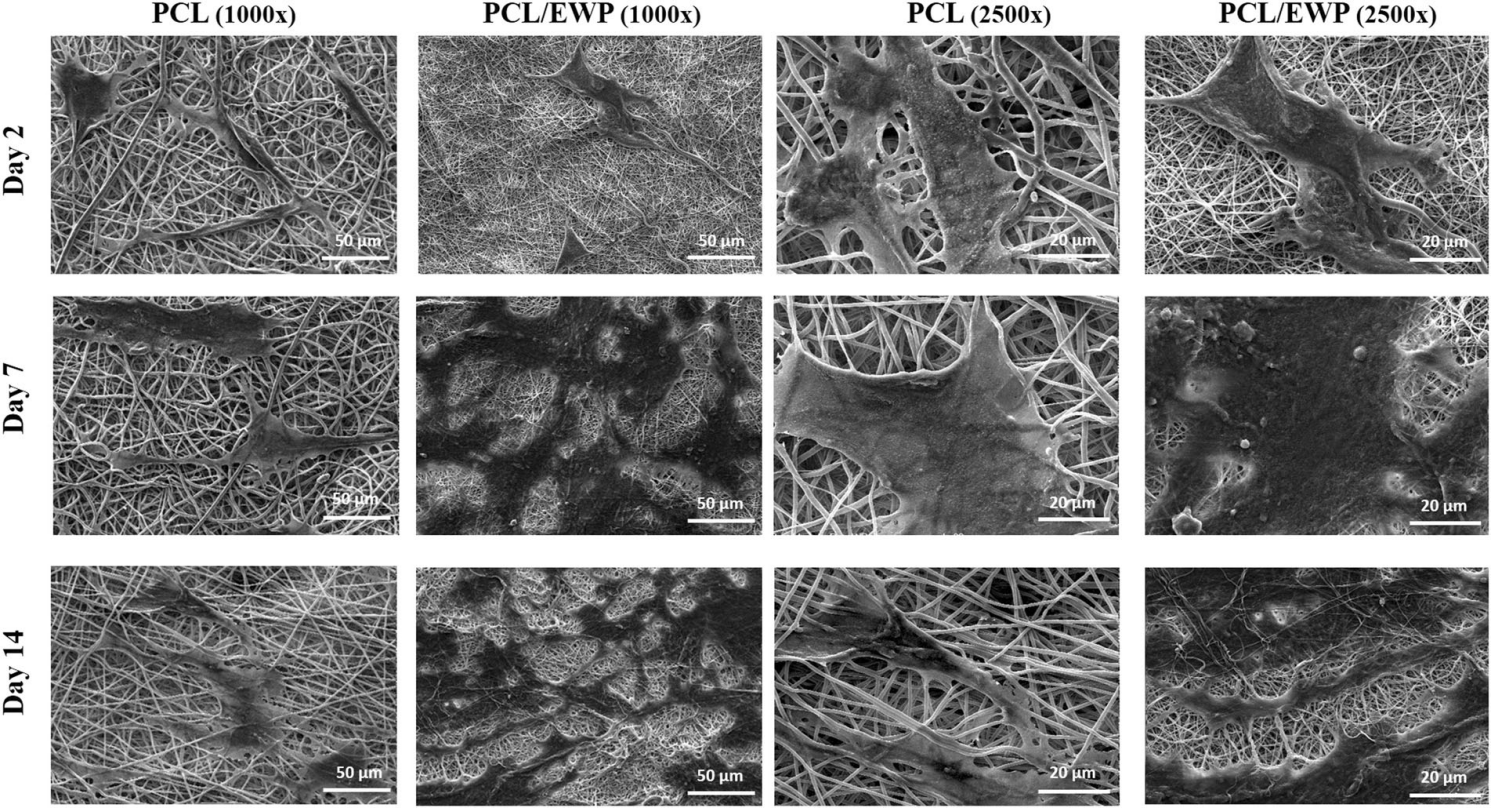
SEM micrographs showing the morphology of the ASCs on neat PCL and PCL/EWP nanofibrous mats at 2, 7, and 14 days of culture (Scale bars; 50 µm,1000× magnification; 20 µm, 2500× magnification)

The influence of EWP on the cell morphology was also evaluated by SEM (Fig. 5). In accordance with the observations of live/dead staining, more uniform cell spreading, and distribution was observed on PCL/EWP mats throughout the culture. On day 2, the area covered by the attached cells were comparable on both surfaces with even more spread cytoskeleton on neat PCL mats (Fig. S3). However, as the culture propagates, ASCs were able to maintain their viability as well as the proliferative state. On day 7, a significant difference was observed at the level of spreading between the two surfaces, cells being spread all over the available surface on PCL/EWP. Similar cellular behavior was also observed on day 14; ASCs being dispersed and spread on PCL/EWP while only a few cells were observed stretched in between the fibers on neat PCL.

**Fig. 6:**
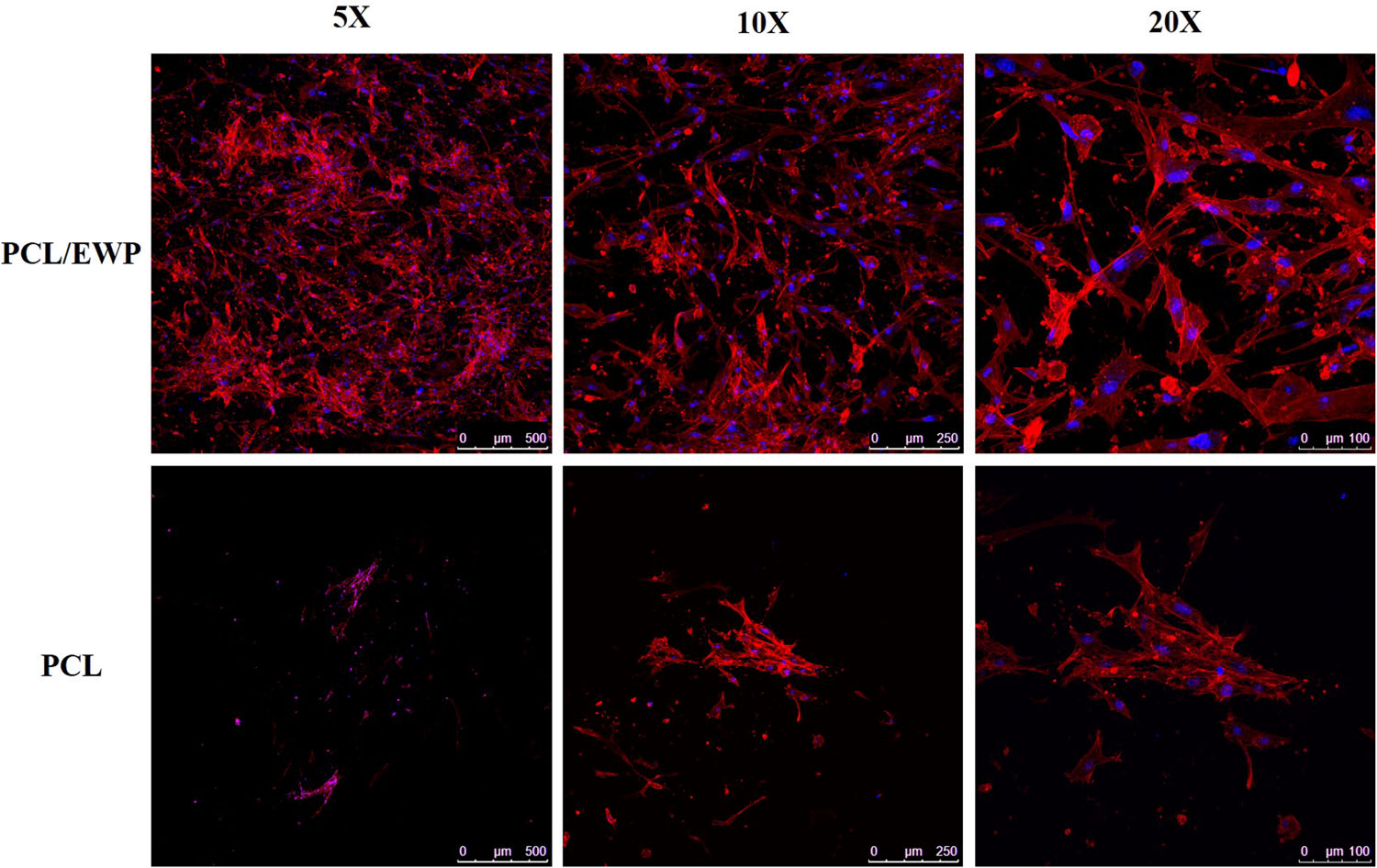
Immunofluorescence micrographs of phalloidin staining (red) of ASCs on neat PCL and PCL/EWP nanofibrous mats at 14 days of culture. Nuclei were counterstained with Draq5 (blue)

The phalloidin staining of actin filaments demonstrated that the cells were uniformly spread and possessed spindle-shaped morphology on the PCL/EWP membranes, whereas they do not exhibit a well-developed cytoskeletal arrangement on neat PCL (Fig. 6). It is known that the organization of actin filaments is largely mediated by the interaction between integrin receptors and matrix proteins [55]. The results are consistent with this claim, as functional groups on PCL/EWP mats can prompt the adsorption of these proteins.

**Fig. 7:**
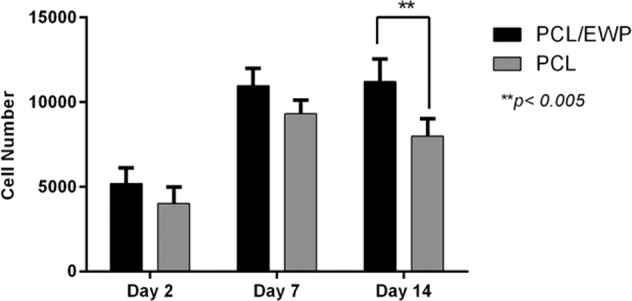
Proliferation of ASCs on neat PCL and PCL/EWP nanofibrous mats determined by Alamar Blue assay (**p < 0.05)

To further confirm the above observations, Alamar Blue assay was performed to quantitate cell proliferation. The number of viable cells was increased both on the PCL/EWP and neat PCL samples during the first 7 days with no statistically significant difference. However, cells proliferated with a much higher rate on PCL/EWP and the viable cell population was significantly higher on PCL/EWP mats compared to the neat PCL on day 14 (Fig. 7). Moreover, a decrease in cell viability observed from day 7 to 14 revealing unfavorable surface properties of neat PCL nanofibers which is in close correlation to the previous reports in the literature [56, 57]. Although the neat PCL nanofibers physically possess the structural aspects of natural ECM to facilitate cell adhesion, their surfaces lack functional active groups such as amine, carboxyl, hydroxyl, and sulfate groups to interact with cells. The incorporation of EWP to the bulk material (through blending) allowed to introduce these essential chemical groups, particularly –NH_2_, which bind integrins via plasma proteins, and to obtain cell-binding amino acid sequences, as well [58, 59]. It has been reported that ovalbumin and ovomucoid have a positive influence on both the proliferation of undifferentiated C2C12 myoblasts and the growth of differentiated C2C12 [60]. Furthermore, ovalbumin has the ability to support both adipogenesis and angiogenesis, as being very similar in amino acid content to bovine serum albumin [59]. Ovalbumin has been also suggested as a scaffold material for bone tissue engineering and able to induce the proliferation and differentiation of preosteoblasts [60]. Herein, we showed the hen egg white holds a synergic combination of these proteins which lead to promote the proliferation and growth of ASCs.

## Conclusions

In conclusion, we have successfully produced nanofibrous mats made of PCL and EWP blend. The addition of EWP to the structure has led to a decrease in fiber diameter, as well as significant changes in surface chemistry and wettability, which are beneficial for cells. Thus, the cell culture studies indicated that the PCL/EWP electrospun mats support the ASC attachment and viability, which supersedes the neat PCL counterparts on day 14 of the culture. Overall, results obtained from the present work indicate that PCL/EWP electrospun mats possess favorable morphological and biochemical properties to serve as an artificial matrix for stem cell growth and can be used as a scaffold for the regeneration of different tissues. The use of EWP as a simple and effective protein supplement to enhance cell spreading and proliferation on PCL nanofibrous scaffolds was shown, which can be further expanded with other synthetic polymers for a variety of tissue engineering applications.

## Supplementary information

Supplementary Information

## References

[1] Nemati S, Kim S-J, Shin YM, Shin H (2019). Current progress in application of polymeric nanofibers to tissue engineering. Nano Converg.

[2] Rahmati M, Mills DK, Urbanska AM, Saeb MR, Venugopal JR, Ramakrishna S et al. Electrospinning for tissue engineering applications. Prog Mater Sci. 2020:100721. 10.1016/j.pmatsci.2020.100721.

[3] Khadka DB, Haynie DT (2012). Protein- and peptide-based electrospun nanofibers in medical biomaterials. Nanomed Nanotechnol Biol Med.

[4] Mondal D, Griffith M, Venkatraman SS (2016). Polycaprolactone-based biomaterials for tissue engineering and drug delivery: current scenario and challenges. Int J Polym Mater Po.

[5] Puppi D, Chiellini F, Piras AM, Chiellini E (2010). Polymeric materials for bone and cartilage repair. Prog Polym Sci.

[6] Jeon H, Lee H, Kim G (2014). A surface-modified poly(e-caprolactone) scaffold comprising variable nanosized surface-roughness using a plasma treatment. Tissue Eng Part C-Me.

[7] Bosworth LA, Hu W, Shi Y, Cartmell SH (2019). Enhancing biocompatibility without compromising material properties: an optimised naoh treatment for electrospun polycaprolactone fibres. J Nanomaterials.

[8] Prabhakaran MP, Venugopal J, Chan CK, Ramakrishna S (2008). Surface modified electrospun nanofibrous scaffolds for nerve tissue engineering. Nanotechnology.

[9] Idini M, Wieringa P, Rocchiccioli S, Nieddu G, Ucciferri N, Formato M (2019). Glycosaminoglycan functionalization of electrospun scaffolds enhances Schwann cell activity. Acta Biomater.

[10] Bual R, Kimura H, Ikegami Y, Shirakigawa N, Ijima H (2018). Fabrication of liver-derived extracellular matrix nanofibers and functional evaluation in in vitro culture using primary hepatocytes. Materialia.

[11] Bhaskar N, Padmavathy N, Jain S, Bose S, Basu B (2016). Modulated in vitro biocompatibility of a unique cross-linked salicylic acid–poly(ε-caprolactone)-based biodegradable polymer. ACS Appl Mater Interfaces.

[12] Zhu Y, Gao C, Liu X, Shen J (2002). Surface modification of polycaprolactone membrane via aminolysis and biomacromolecule immobilization for promoting cytocompatibility of human endothelial cells. Biomacromolecules.

[13] Ravikrishnan A, Ozdemir T, Bah M, Baskerville KA, Shah SI, Rajasekaran AK (2016). Regulation of epithelial-to-mesenchymal transition using biomimetic fibrous scaffolds. ACS Appl Mater interfaces.

[14] Wang Z, Wang Z, Lu WW, Zhen W, Yang D, Peng S (2017). Novel biomaterial strategies for controlled growth factor delivery for biomedical applications. Npg Asia Mater.

[15] Bucci R, Vaghi F, Erba E, Romanelli A, Gelmi ML, Clerici F (2020). Peptide grafting strategies before and after electrospinning of nanofibers. Acta Biomater.

[16] Irani S, Honarpardaz A, Choubini N, Pezeshki-Modaress M, Zandi M. Chondro-inductive nanofibrous scaffold based gelatin/polyvinyl alcohol/chondroitin sulfate for cartilage tissue engineering. 2020;31:1395–402. 10.1002/pat.4869.

[17] Ranjbar-Mohammadi M, Mousavi E, Mostakhdem Hashemi M, Abbasian M, Asadi J, Esmaili E (2020). Efficient co-cultivation of human fibroblast cells (HFCs) and adipose-derived stem cells (ADSs) on gelatin/PLCL nanofiber. IET Nanobiotechnol.

[18] Sadeghi A, Zandi M, Pezeshki-Modaress M, Rajabi S (2019). Tough, hybrid chondroitin sulfate nanofibers as a promising scaffold for skin tissue engineering. Int J Biol Macromol.

[19] Perez-Puyana V, Jiménez-Rosado M, Guerrero A, Romero A (2020). Anisotropic properties of PCL/gelatin scaffolds obtained via electrospinning. Int J Fract.

[20] Fu W, Liu Z, Feng B, Hu R, He X, Wang H (2014). Electrospun gelatin/PCL and collagen/PLCL scaffolds for vascular tissue engineering. Int J Nanomed.

[21] Ren K, Wang Y, Sun T, Yue W, Zhang H (2017). Electrospun PCL/gelatin composite nanofiber structures for effective guided bone regeneration membranes. Mater Sci Eng C Mater Biol Appl.

[22] Schnell E, Klinkhammer K, Balzer S, Brook G, Klee D, Dalton P (2007). Guidance of glial cell migration and axonal growth on electrospun nanofibers of poly-epsilon-caprolactone and a collagen/poly-epsilon-caprolactone blend. Biomaterials.

[23] Bockelmann J, Klinkhammer K, von Holst A, Seiler N, Faissner A, Brook GA (2011). Functionalization of electrospun poly(epsilon-caprolactone) fibers with the extracellular matrix-derived peptide GRGDS improves guidance of schwann cell migration and axonal growth. Tissue Eng Part A.

[24] Strixner T, Kulozik U. 7 - Egg proteins. In: Phillips GO, Williams PA, eds. Handbook of Food Proteins. Woodhead Publishing; 2011. pp. 150–209.

[25] Fujita H, Yokoyama K, Yoshikawa M (2000). Classification and antihypertensive activity of angiotensin I-converting enzyme inhibitory peptides derived from food proteins. J Food Sci.

[26] Miguel M, Aleixandre A (2006). Anti hypertensive peptides derived from egg proteins. J Nutr.

[27] Jalili-Firoozinezhad S, Filippi M, Mohabatpour F, Letourneur D, Scherberich A (2020). Chicken egg white: Hatching of a new old biomaterial. Mater Today.

[28] Superti F, Ammendolia MG, Berlutti F, Valenti P, Huopalahti R, López-Fandiño R, Anton M, Schade R (2007). Ovotransferrin. Bioactive egg compounds.

[29] Watanabe K, Tsuge Y, Shimoyamada M, Ogama N, Ebina T (1998). Antitumor effects of pronase-treated fragments, glycopeptides, from ovomucin in hen egg white in a double grafted tumor system. J Agr Food Chem.

[30] Sugahara T, Murakami F, Yamada Y, Sasaki T (2000). The mode of actions of lysozyme as an immunoglobulin production stimulating factor. Bba-Gen Subj.

[31] Pacor S, Gagliardi R, Di Daniel E, Vadori M, Sava G (1999). In vitro down regulation of ICAM-1 and E-cadherin and in vivo reduction of lung metastases of TS/A adenocarcinoma by a lysozyme derivative. Int J Mol Med.

[32] Sava G (1989). Reduction of B16 melanoma metastases by oral administration of egg-white lysozyme. Cancer Chemother Pharm.

[33] Nojima T, Iyoda T. Egg white-based strong hydrogel via ordered protein condensation. Npg Asia Mater. 2018;10:e460. 10.1038/am.2017.219.

[34] Laemmli UK (1970). Cleavage of structural proteins during the assembly of the head of bacteriophage T4. Nature.

[35] Sartika D, Wang CH, Wang DH, Cherng JH, Chang SJ, Fan GY et al. Human adipose-derived mesenchymal stem cells-incorporated silk fibroin as a potential bio-scaffold in guiding bone regeneration. Polymers. 2020;12. 10.3390/polym12040853. 10.3390/polym12040853 PMC7240549 32272682

[36] Zhou Y, Xia X, Yang E, Wang Y, Marra KG, Ethier CR (2020). Adipose-derived stem cells integrate into trabecular meshwork with glaucoma treatment potential. FASEB J.

[37] Shafaei H, Kalarestaghi H (2020). Adipose-derived stem cells: an appropriate selection for osteogenic differentiation. J Cell Physiol.

[38] Kokubu S, Inaki R, Hoshi K, Hikita A (2020). Adipose-derived stem cells improve tendon repair and prevent ectopic ossification in tendinopathy by inhibiting inflammation and inducing neovascularization in the early stage of tendon healing. Regen Ther.

[39] Huri PY (2015). Effect of culture conditions on the multinucleation of human adipose-derived stem cells. J Biomater Tiss Eng.

[40] Miguel M, Manso MA, Lopez-Fandino R, Ramos M (2005). Comparative study of egg white proteins from different species by chromatographic and electrophoretic methods. Eur Food Res Technol.

[41] Jhala D, Rather H, Kedaria D, Shah J, Singh S, Vasita R (2019). Biomimetic polycaprolactone-chitosan nanofibrous substrate influenced cell cycle and ECM secretion affect cellular uptake of nanoclusters. Bioact Mater.

[42] Bhardwaj N, Kundu SC (2010). Electrospinning: a fascinating fiber fabrication technique. Biotechnol Adv.

[43] Deitzel JM, Kleinmeyer J, Harris D, Beck Tan NC (2001). The effect of processing variables on the morphology of electrospun nanofibers and textiles. Polymer.

[44] Tiwari AP, Joshi MK, Kim JI, Unnithan AR, Lee J, Park CH (2016). Bimodal fibrous structures for tissue engineering: Fabrication, characterization and in vitro biocompatibility. J Colloid Interface Sci.

[45] He W, Yong T, Teo WE, Ma ZW, Ramakrishna S (2005). Fabrication and endothelialization of collagen-blended biodegradable polymer nanofibers: Potential vascular graft for blood vessel tissue engineering. Tissue Eng.

[46] Phillipson K, Hay JN, Jenkins MJ (2014). Thermal analysis FTIR spectroscopy of poly(epsilon-caprolactone). Thermochim Acta.

[47] Barth A (2007). Infrared spectroscopy of proteins. Bba-Bioenerg.

[48] Keselowsky BG, Collard DM, Garcia AJ (2004). Surface chemistry modulates focal adhesion composition and signaling through changes in integrin binding. Biomaterials.

[49] Griffin MF, Ibrahim A, Seifalian AM, Butler PEM, Kalaskar DM, Ferretti P (2017). Chemical group-dependent plasma polymerisation preferentially directs adipose stem cell differentiation towards osteogenic or chondrogenic lineages. Acta Biomater.

[50] Heydari Z, Mohebbi-Kalhori D, Afarani MS (2017). Engineered electrospun polycaprolactone (PCL)/octacalcium phosphate (OCP) scaffold for bone tissue engineering. Mat Sci Eng C-Mater.

[51] Chakrapani VY, Gnanamani A, Giridev VR, Madhusoothanan M, Sekaran G (2012). Electrospinning of type I collagen and PCL nanofibers using acetic acid. J Appl Polym Sci.

[52] Sahin YM, Su S, Ozbek B, Yücel S, Pinar O, Kazan D (2018). Production and characterization of electrospun fish sarcoplasmic protein based nanofibers. J Food Eng.

[53] Aguirre-Chagala YE, Altuzar V, Leon-Sarabia E, Tinoco-Magana JC, Yanez-Limon JM, Mendoza-Barrera C (2017). Physicochemical properties of polycaprolactone/collagen/elastin nanofibers fabricated by electrospinning. Mater Sci Eng C Mater Biol Appl.

[54] Roozbahani F, Sultana N, Almasi D, Naghizadeh F (2015). Effects of chitosan concentration on the protein release behaviour of electrospun poly(-caprolactone)/chitosan nanofibers. J Nanomaterials.

[55] Hersel U, Dahmen C, Kessler H (2003). RGD modified polymers: biomaterials for stimulated cell adhesion and beyond. Biomaterials.

[56] Abbasi N, Soudi S, Hayati-Roodbari N, Dodel M, Soleimani M (2014). The effects of plasma treated electrospun nanofibrous poly (epsilon-caprolactone) scaffolds with different orientations on mouse embryonic stem cell proliferation. Cell J.

[57] Li JD, Chen ML, Wei XY, Hao YS, Wang JM. Evaluation of 3D-printed polycaprolactone scaffolds coated with freeze-dried platelet-rich plasma for bone regeneration. Materials. 2017;10:831. 10.3390/ma10070831. 10.3390/ma10070831 PMC5551874 28773189

[58] Zou C, Kobayashi K, Kato A (1991). Effects of chicken egg-white on the proliferation and neurite outgrowth of mammalian-cells. J Agr Food Chem.

[59] Luo BW, Choong C (2015). Porous ovalbumin scaffolds with tunable properties: a resource-efficient biodegradable material for tissue engineering applications. J Biomater Appl.

[60] Farrar G, Barone J, Morgan A (2010). Ovalbumin-based porous scaffolds for bone tissue regeneration. J Tissue Eng.

