# Supplementary material for: Facile modification of polycaprolactone nanofibers with egg white protein
Source: J Mater Sci Mater Med. 2021 Mar 24;32(4):34. doi: 10.1007/s10856-021-06505-x (PMC7990845; doi:10.1007/s10856-021-06505-x)
Supplement: Supplementary file 1 — Supplementary Information [file 10856_2021_6505_MOESM1_ESM.pdf]

Facile Modification of Polycaprolactone Nanofibers with Egg White Protein

Nergis Zeynep Renkler[1*], Emre Ergene[2], Seyda Gokyer[2], Merve Tuzlakoglu Ozturk[3],

Pinar Yilgor Huri[2], Kadriye Tuzlakoglu[1]

[1] Yalova University, Department of Polymer Engineering, 77200, Yalova, Turkey

[2] Ankara University, Department of Biomedical Engineering, Ankara, Turkey

[3] Gebze Technical University, Department of Molecular Biology and Genetics, Kocaeli, Turkey

*Correspondence to: Nergis Zeynep Renkler (E-mail: *nzrenkler@gmail.com*)

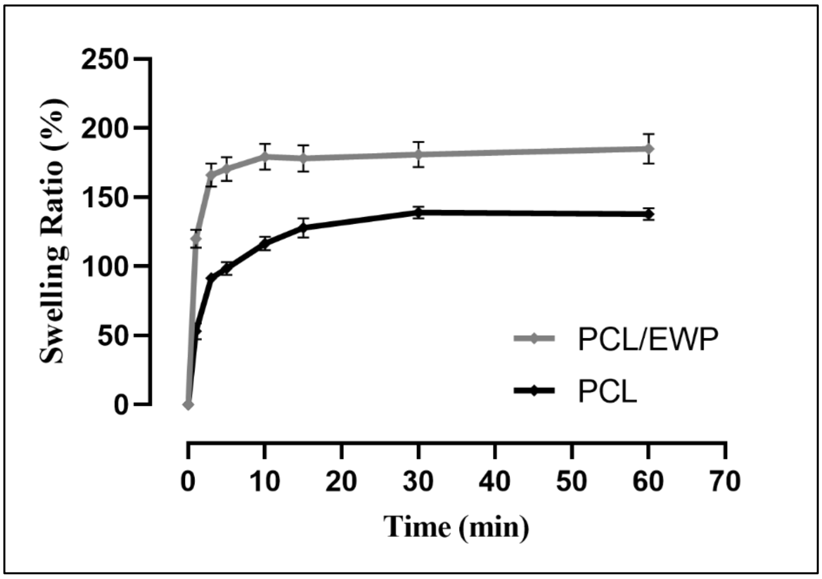

Fig. S1 Swelling ratio of PCL/EWP and PCL mats over time (n=4)

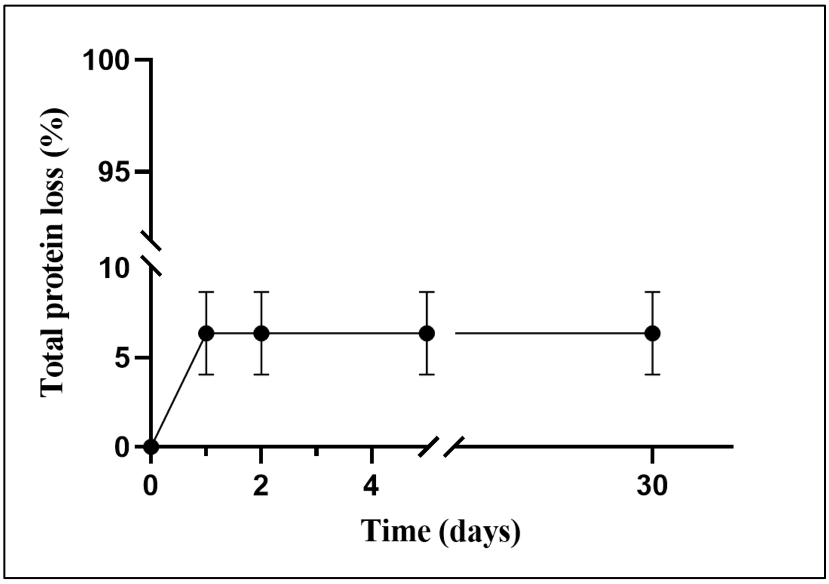

Fig. S2 The amount of protein loss from the PCL/EWP mats over time (n=4)

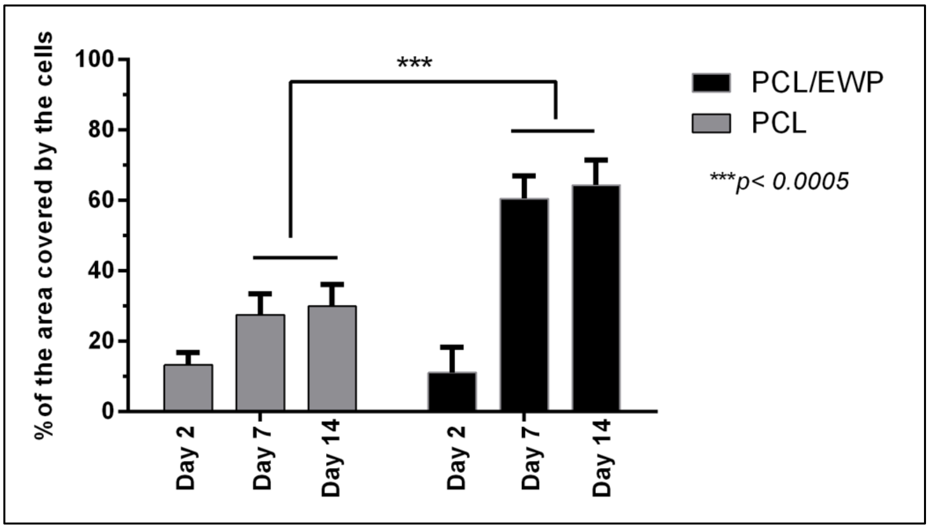

Fig. S3 Area covered by the ASCs on PCL/EWP and PCL mats on days 2, 7, and 14
